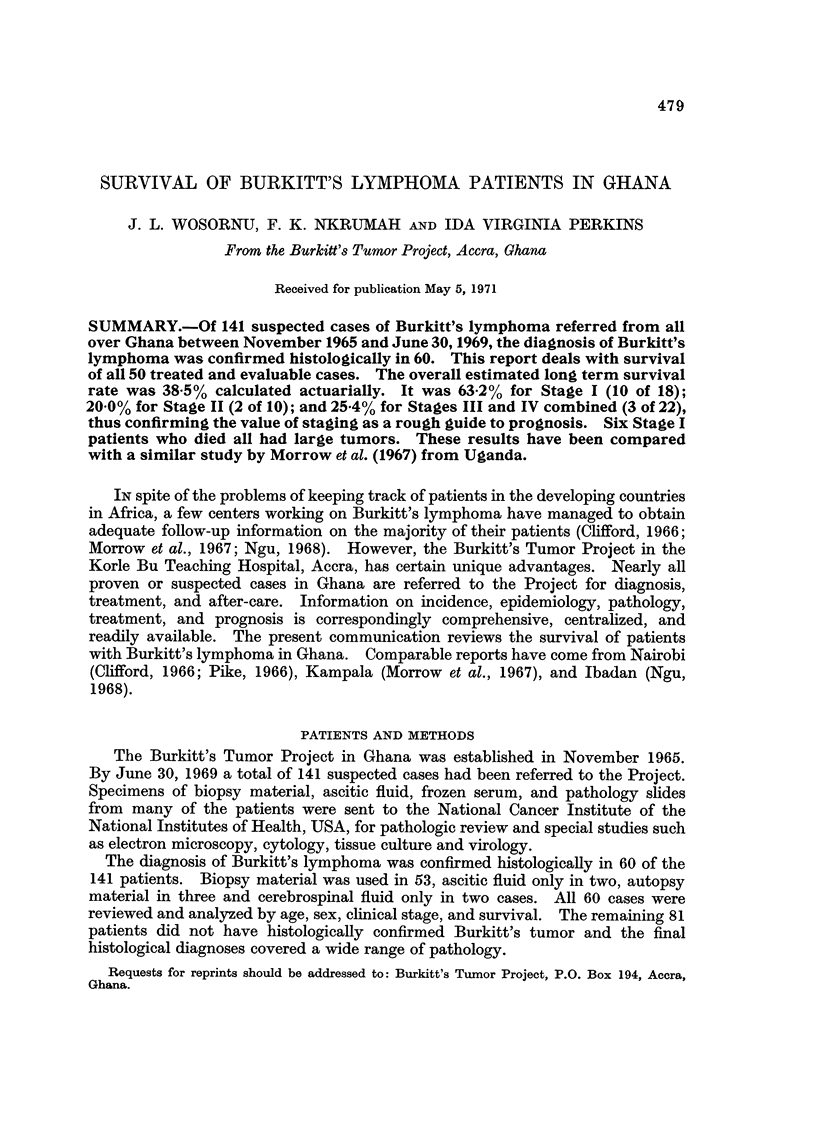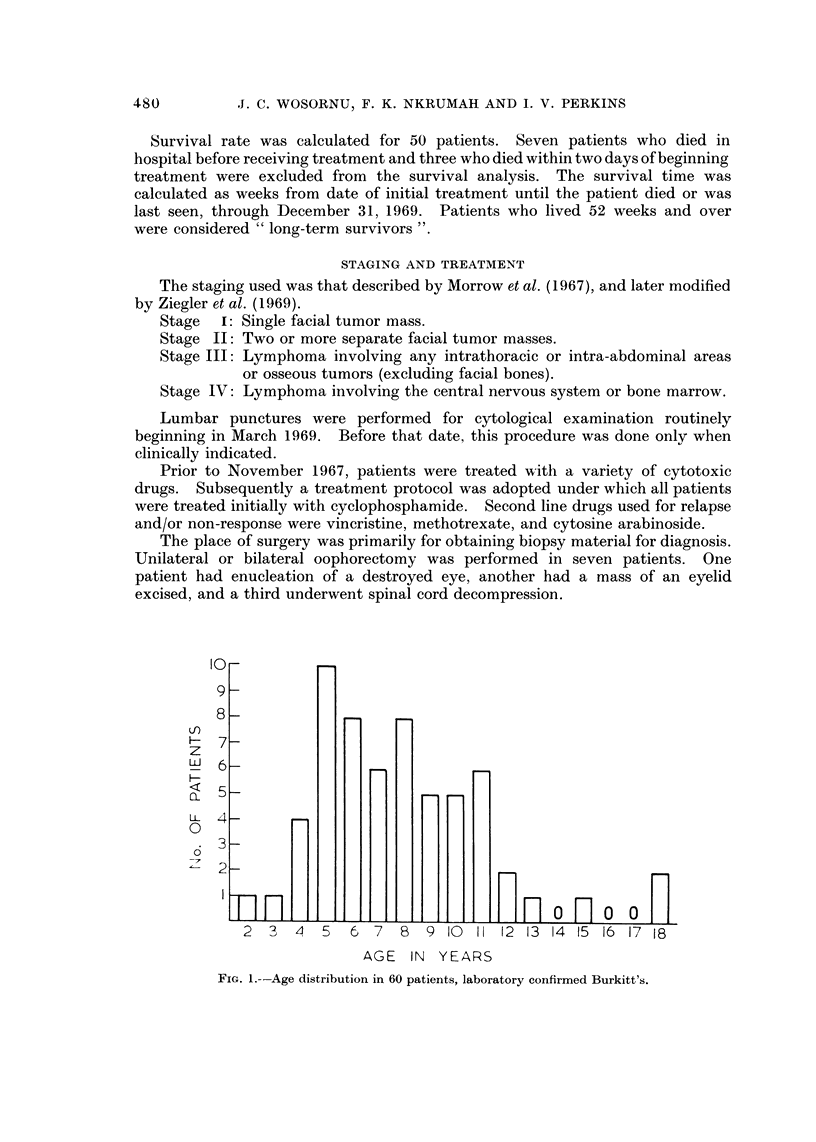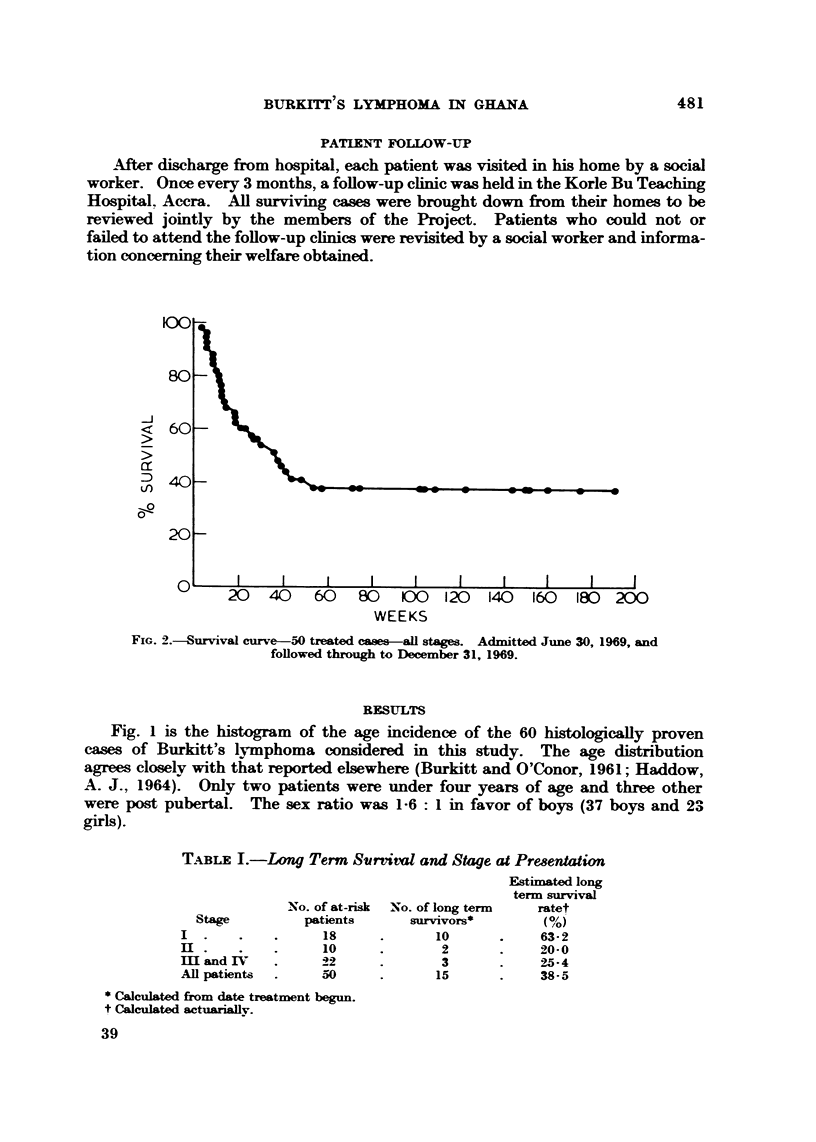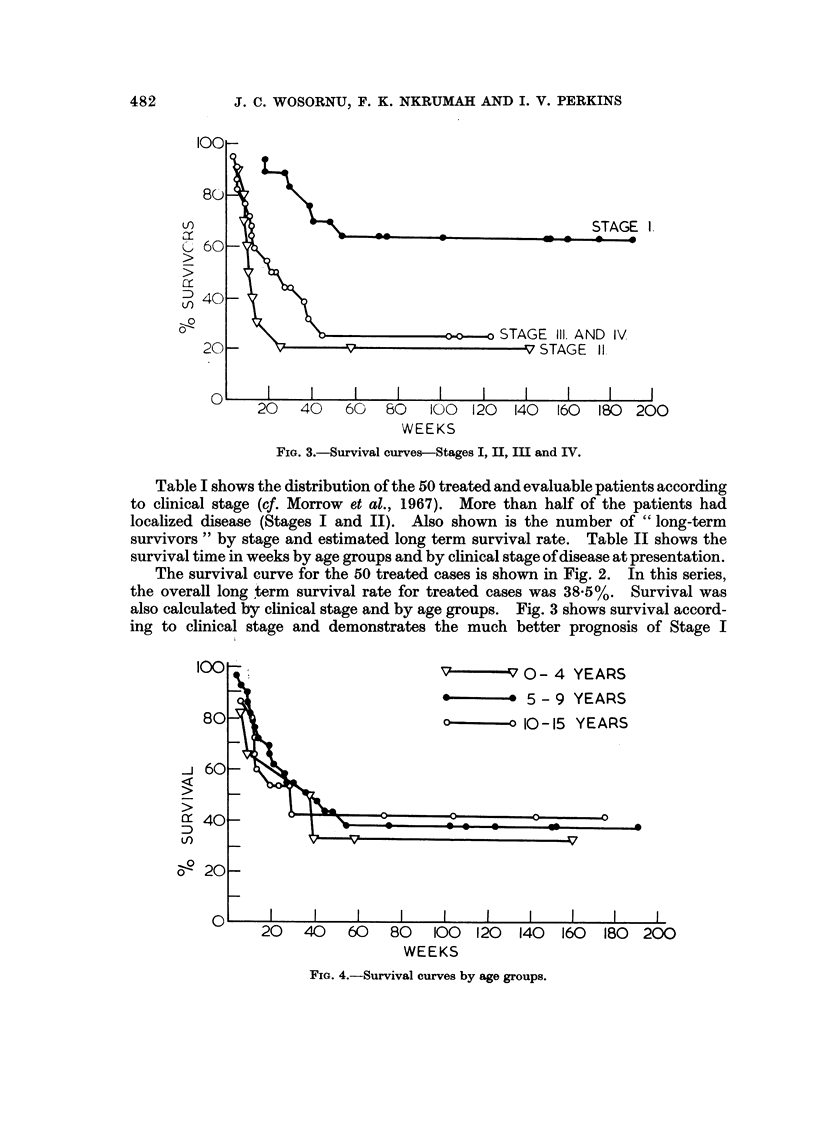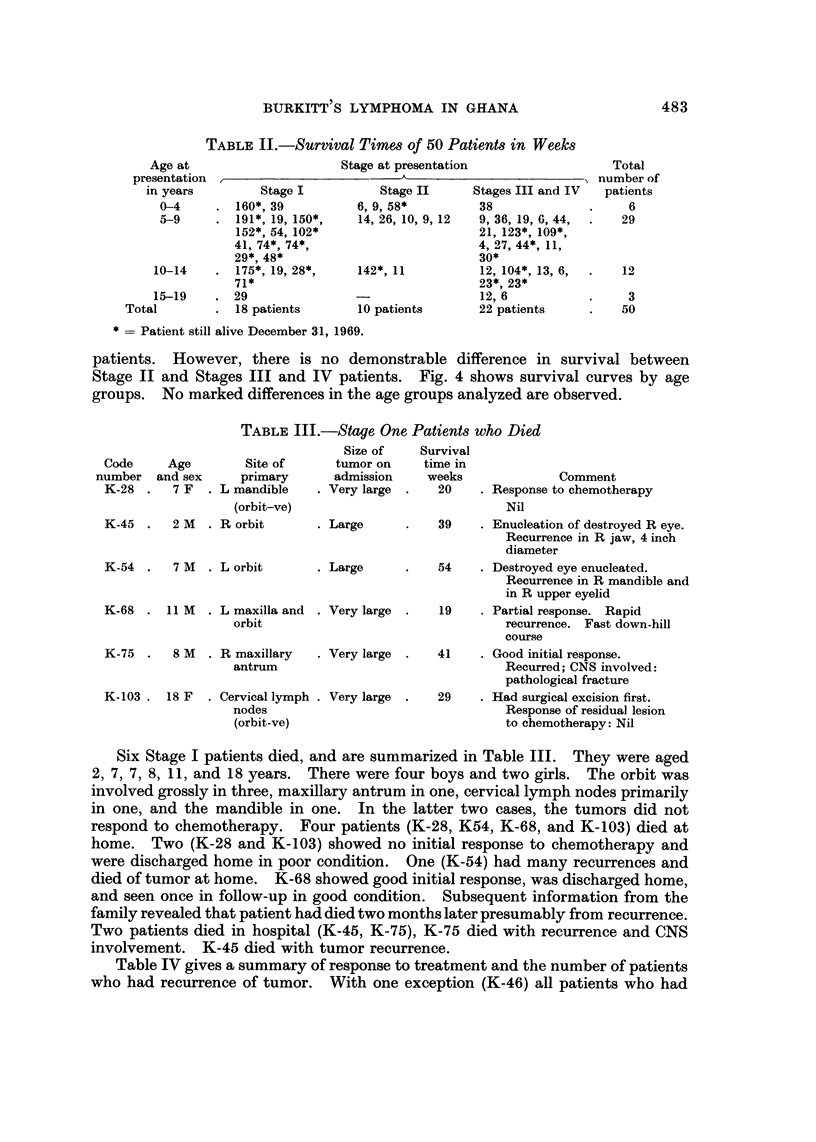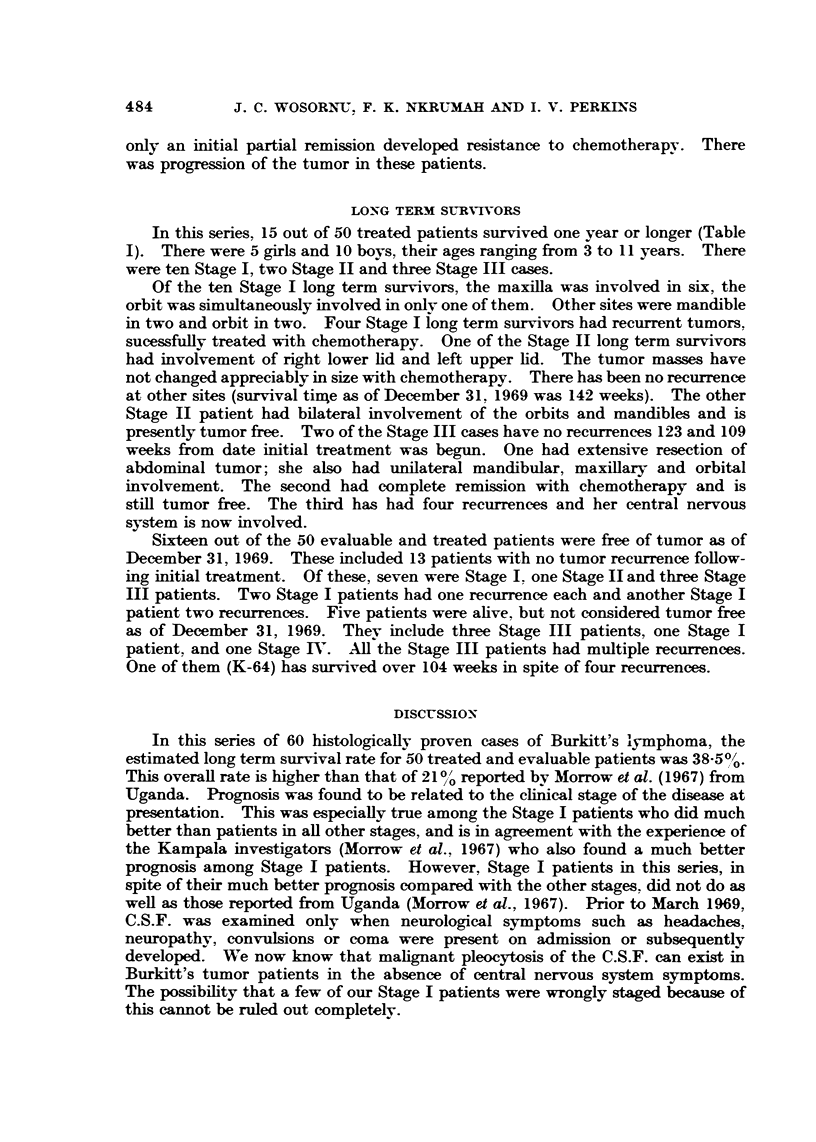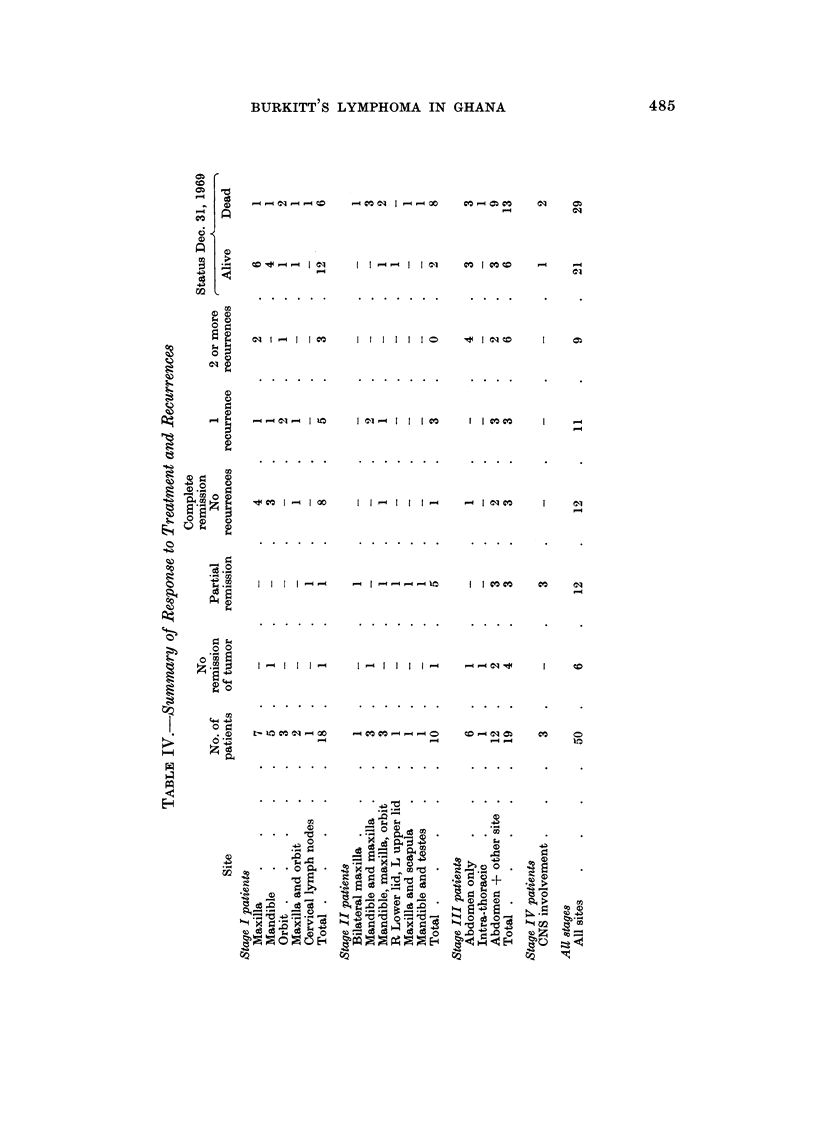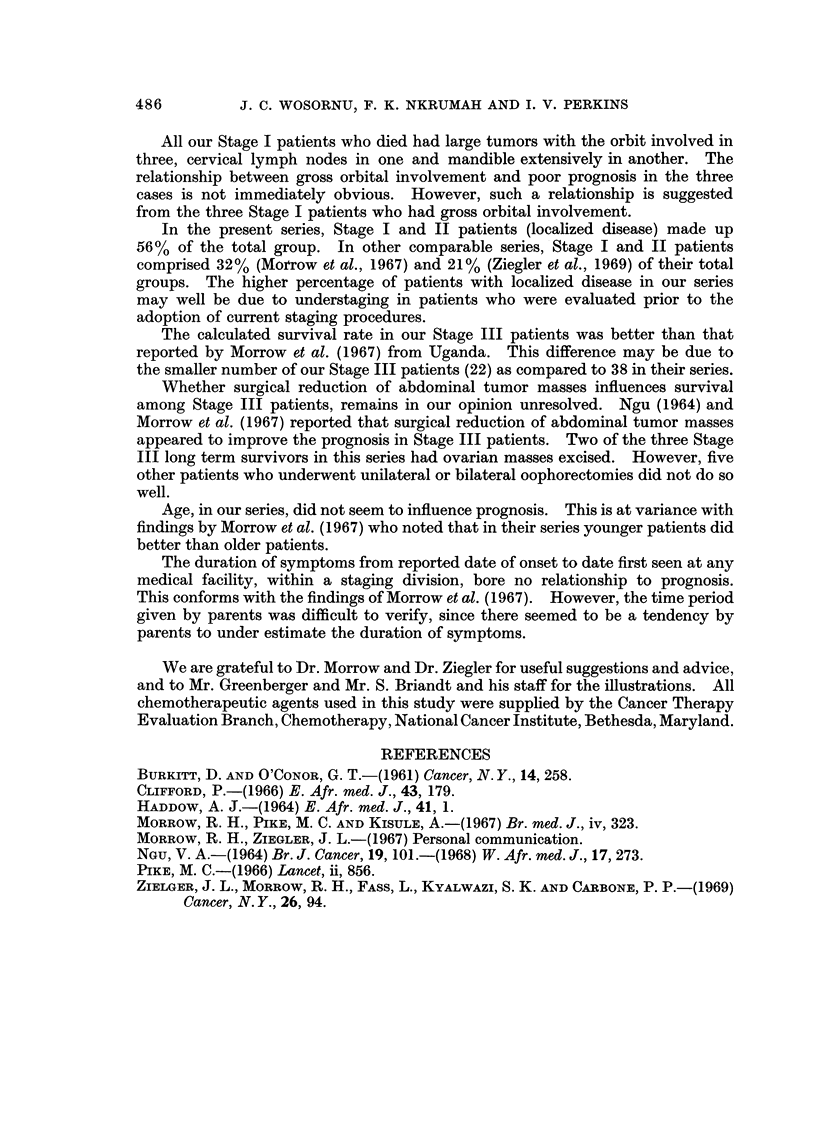# Survival of Burkitt's Lymphoma Patients in Ghana

**DOI:** 10.1038/bjc.1971.60

**Published:** 1971-09

**Authors:** J. L. Wosornu, F. K. Nkrumah, Ida Virginia Perkins

## Abstract

Of 141 suspected cases of Burkitt's lymphoma referred from all over Ghana between November 1965 and June 30, 1969, the diagnosis of Burkitt's lymphoma was confirmed histologically in 60. This report deals with survival of all 50 treated and evaluable cases. The overall estimated long term survival rate was 38·5% calculated actuarially. It was 63·2% for Stage I (10 of 18); 20·0% for Stage II (2 of 10); and 25·4% for Stages III and IV combined (3 of 22), thus confirming the value of staging as a rough guide to prognosis. Six Stage I patients who died all had large tumors. These results have been compared with a similar study by Morrow *et al.* (1967) from Uganda.


### SURVIVAL OF BURKITT'S LYMPHOMA PATIENTS IN GHANA

J. L. WOSORNU, F. K. NKRUMAH AND IDA VIRGINIA PERKINS

*From the Burkitt's Tumor Project, Accra, Ghana*

Received for publication May 5, 1971

**SUMMARY.—Of 141 suspected cases of Burkitt's lymphoma referred from all over Ghana between November 1965 and June 30, 1969, the diagnosis of Burkitt's lymphoma was confirmed histologically in 60. This report deals with survival of all 50 treated and evaluable cases. The overall estimated long term survival rate was 38·5% calculated actuarially. It was 63·2% for Stage I (10 of 18); 20·0% for Stage II (2 of 10); and 25·4% for Stages III and IV combined (3 of 22), thus confirming the value of staging as a rough guide to prognosis. Six Stage I patients who died all had large tumors. These results have been compared with a similar study by Morrow *et al.* (1967) from Uganda.**

In spite of the problems of keeping track of patients in the developing countries in Africa, a few centers working on Burkitt's lymphoma have managed to obtain adequate follow-up information on the majority of their patients (Clifford, 1966; Morrow *et al.*, 1967; Ngu, 1968). However, the Burkitt's Tumor Project in the Korle Bu Teaching Hospital, Accra, has certain unique advantages. Nearly all proven or suspected cases in Ghana are referred to the Project for diagnosis, treatment, and after-care. Information on incidence, epidemiology, pathology, treatment, and prognosis is correspondingly comprehensive, centralized, and readily available. The present communication reviews the survival of patients with Burkitt's lymphoma in Ghana. Comparable reports have come from Nairobi (Clifford, 1966; Pike, 1966), Kampala (Morrow *et al.*, 1967), and Ibadan (Ngu, 1968).

### PATIENTS AND METHODS

The Burkitt's Tumor Project in Ghana was established in November 1965. By June 30, 1969 a total of 141 suspected cases had been referred to the Project. Specimens of biopsy material, ascitic fluid, frozen serum, and pathology slides from many of the patients were sent to the National Cancer Institute of the National Institutes of Health, USA, for pathologic review and special studies such as electron microscopy, cytology, tissue culture and virology.

The diagnosis of Burkitt's lymphoma was confirmed histologically in 60 of the 141 patients. Biopsy material was used in 53, ascitic fluid only in two, autopsy material in three and cerebrospinal fluid only in two cases. All 60 cases were reviewed and analyzed by age, sex, clinical stage, and survival. The remaining 81 patients did not have histologically confirmed Burkitt's tumor and the final histological diagnoses covered a wide range of pathology.

Requests for reprints should be addressed to: Burkitt's Tumor Project, P.O. Box 194, Accra, Ghana.

Survival rate was calculated for 50 patients. Seven patients who died in hospital before receiving treatment and three who died within two days of beginning treatment were excluded from the survival analysis. The survival time was calculated as weeks from date of initial treatment until the patient died or was last seen, through December 31, 1969. Patients who lived 52 weeks and over were considered " long-term survivors ".

### STAGING AND TREATMENT

The staging used was that described by Morrow *et al.* (1967), and later modified by Ziegler *et al.* (1969).

Stage I: Single facial tumor mass.

Stage II: Two or more separate facial tumor masses.

Stage III: Lymphoma involving any intrathoracic or intra-abdominal areas or osseous tumors (excluding facial bones).

Stage IV: Lymphoma involving the central nervous system or bone marrow.

Lumbar punctures were performed for cytological examination routinely beginning in March 1969. Before that date, this procedure was done only when clinically indicated.

Prior to November 1967, patients were treated with a variety of cytotoxic drugs. Subsequently a treatment protocol was adopted under which all patients were treated initially with cyclophosphamide. Second line drugs used for relapse and/or non-response were vincristine, methotrexate, and cytosine arabinoside.

The place of surgery was primarily for obtaining biopsy material for diagnosis. Unilateral or bilateral oophorectomy was performed in seven patients. One patient had enucleation of a destroyed eye, another had a mass of an eyelid excised, and a third underwent spinal cord decompression.

FIG. 1.—Age distribution in 60 patients, laboratory confirmed Burkitt's.

PATIENT FOLLOW-UP

After discharge from hospital, each patient was visited in his home by a social worker. Once every 3 months, a follow-up clinic was held in the Korle Bu Teaching Hospital, Accra. All surviving cases were brought down from their homes to be reviewed jointly by the members of the Project. Patients who could not or failed to attend the follow-up clinics were revisited by a social worker and information concerning their welfare obtained.

FIG. 2.—Survival curve—50 treated cases—all stages. Admitted June 30, 1969, and followed through to December 31, 1969.

RESULTS

Fig. 1 is the histogram of the age incidence of the 60 histologically proven cases of Burkitt's lymphoma considered in this study. The age distribution agrees closely with that reported elsewhere (Burkitt and O'Conor, 1961; Haddow, A. J., 1964). Only two patients were under four years of age and three other were post pubertal. The sex ratio was 1·6 : 1 in favor of boys (37 boys and 23 girls).

TABLE I.—*Long Term Survival and Stage at Presentation*

| Stage | No. of at-risk patients | No. of long term survivors* | Estimated long term survival rate† (%) |
|---|---|---|---|
| I | 18 | 10 | 63·2 |
| II | 10 | 2 | 20·0 |
| III and IV | 22 | 3 | 25·4 |
| All patients | 50 | 15 | 38·5 |

* Calculated from date treatment begun.

† Calculated actuarially.

FIG. 3.—Survival curves—Stages I, II, III and IV.

Table I shows the distribution of the 50 treated and evaluable patients according to clinical stage (*cf.* Morrow *et al.*, 1967). More than half of the patients had localized disease (Stages I and II). Also shown is the number of " long-term survivors " by stage and estimated long term survival rate. Table II shows the survival time in weeks by age groups and by clinical stage of disease at presentation.

The survival curve for the 50 treated cases is shown in Fig. 2. In this series, the overall long term survival rate for treated cases was 38·5%. Survival was also calculated by clinical stage and by age groups. Fig. 3 shows survival according to clinical stage and demonstrates the much better prognosis of Stage I

FIG. 4.—Survival curves by age groups.

TABLE II.—*Survival Times of 50 Patients in Weeks*

| Age at presentation in years | Stage at presentation: Stage I | Stage II | Stages III and IV | Total number of patients |
|---|---|---|---|---|
| 0–4 | 160*, 39 | 6, 9, 58* | 38 | 6 |
| 5–9 | 191*, 19, 150*, 152*, 54, 102* 41, 74*, 74*, 29*, 48* | 14, 26, 10, 9, 12 | 9, 36, 19, 6, 44, 21, 123*, 109*, 4, 27, 44*, 11, 30* | 29 |
| 10–14 | 175*, 19, 28*, 71* | 142*, 11 | 12, 104*, 13, 6, 23*, 23* | 12 |
| 15–19 | 29 | — | 12, 6 | 3 |
| Total | 18 patients | 10 patients | 22 patients | 50 |

* = Patient still alive December 31, 1969.

patients. However, there is no demonstrable difference in survival between Stage II and Stages III and IV patients. Fig. 4 shows survival curves by age groups. No marked differences in the age groups analyzed are observed.

TABLE III.—*Stage One Patients who Died*

| Code number | Age and sex | Site of primary | Size of tumor on admission | Survival time in weeks | Comment |
|---|---|---|---|---|---|
| K-28 | 7 F | L mandible (orbit–ve) | Very large | 20 | Response to chemotherapy Nil |
| K-45 | 2 M | R orbit | Large | 39 | Enucleation of destroyed R eye. Recurrence in R jaw, 4 inch diameter |
| K-54 | 7 M | L orbit | Large | 54 | Destroyed eye enucleated. Recurrence in R mandible and in R upper eyelid |
| K-68 | 11 M | L maxilla and orbit | Very large | 19 | Partial response. Rapid recurrence. Fast down-hill course |
| K-75 | 8 M | R maxillary antrum | Very large | 41 | Good initial response. Recurred; CNS involved: pathological fracture |
| K-103 | 18 F | Cervical lymph nodes (orbit-ve) | Very large | 29 | Had surgical excision first. Response of residual lesion to chemotherapy: Nil |

Six Stage I patients died, and are summarized in Table III. They were aged 2, 7, 7, 8, 11, and 18 years. There were four boys and two girls. The orbit was involved grossly in three, maxillary antrum in one, cervical lymph nodes primarily in one, and the mandible in one. In the latter two cases, the tumors did not respond to chemotherapy. Four patients (K-28, K54, K-68, and K-103) died at home. Two (K-28 and K-103) showed no initial response to chemotherapy and were discharged home in poor condition. One (K-54) had many recurrences and died of tumor at home. K-68 showed good initial response, was discharged home, and seen once in follow-up in good condition. Subsequent information from the family revealed that patient had died two months later presumably from recurrence. Two patients died in hospital (K-45, K-75), K-75 died with recurrence and CNS involvement. K-45 died with tumor recurrence.

Table IV gives a summary of response to treatment and the number of patients who had recurrence of tumor. With one exception (K-46) all patients who had only an initial partial remission developed resistance to chemotherapy. There was progression of the tumor in these patients.

### LONG TERM SURVIVORS

In this series, 15 out of 50 treated patients survived one year or longer (Table I). There were 5 girls and 10 boys, their ages ranging from 3 to 11 years. There were ten Stage I, two Stage II and three Stage III cases.

Of the ten Stage I long term survivors, the maxilla was involved in six, the orbit was simultaneously involved in only one of them. Other sites were mandible in two and orbit in two. Four Stage I long term survivors had recurrent tumors, sucessfully treated with chemotherapy. One of the Stage II long term survivors had involvement of right lower lid and left upper lid. The tumor masses have not changed appreciably in size with chemotherapy. There has been no recurrence at other sites (survival time as of December 31, 1969 was 142 weeks). The other Stage II patient had bilateral involvement of the orbits and mandibles and is presently tumor free. Two of the Stage III cases have no recurrences 123 and 109 weeks from date initial treatment was begun. One had extensive resection of abdominal tumor; she also had unilateral mandibular, maxillary and orbital involvement. The second had complete remission with chemotherapy and is still tumor free. The third has had four recurrences and her central nervous system is now involved.

Sixteen out of the 50 evaluable and treated patients were free of tumor as of December 31, 1969. These included 13 patients with no tumor recurrence following initial treatment. Of these, seven were Stage I, one Stage II and three Stage III patients. Two Stage I patients had one recurrence each and another Stage I patient two recurrences. Five patients were alive, but not considered tumor free as of December 31, 1969. They include three Stage III patients, one Stage I patient, and one Stage IV. All the Stage III patients had multiple recurrences. One of them (K-64) has survived over 104 weeks in spite of four recurrences.

### DISCUSSION

In this series of 60 histologically proven cases of Burkitt's lymphoma, the estimated long term survival rate for 50 treated and evaluable patients was 38·5%. This overall rate is higher than that of 21% reported by Morrow *et al.* (1967) from Uganda. Prognosis was found to be related to the clinical stage of the disease at presentation. This was especially true among the Stage I patients who did much better than patients in all other stages, and is in agreement with the experience of the Kampala investigators (Morrow *et al.*, 1967) who also found a much better prognosis among Stage I patients. However, Stage I patients in this series, in spite of their much better prognosis compared with the other stages, did not do as well as those reported from Uganda (Morrow *et al.*, 1967). Prior to March 1969, C.S.F. was examined only when neurological symptoms such as headaches, neuropathy, convulsions or coma were present on admission or subsequently developed. We now know that malignant pleocytosis of the C.S.F. can exist in Burkitt's tumor patients in the absence of central nervous system symptoms. The possibility that a few of our Stage I patients were wrongly staged because of this cannot be ruled out completely.

TABLE IV.—*Summary of Response to Treatment and Recurrences*

| Site | No. of patients | No remission of tumor | Partial remission | Complete remission No recurrences | Complete remission 1 recurrence | Complete remission 2 or more recurrences | Status Dec. 31, 1969 Alive | Status Dec. 31, 1969 Dead |
|---|---|---|---|---|---|---|---|---|
| *Stage I patients* | | | | | | | | |
| Maxilla | 7 | – | – | 4 | 1 | 2 | 6 | 1 |
| Mandible | 5 | 1 | – | 3 | 1 | – | 4 | 1 |
| Orbit | 3 | – | – | – | 2 | 1 | 1 | 2 |
| Maxilla and orbit | 2 | – | – | 1 | 1 | – | 1 | 1 |
| Cervical lymph nodes | 1 | – | 1 | – | – | – | – | 1 |
| Total | 18 | 1 | 1 | 8 | 5 | 3 | 12 | 6 |
| *Stage II patients* | | | | | | | | |
| Bilateral maxilla | 1 | – | 1 | – | – | – | – | 1 |
| Mandible and maxilla | 3 | 1 | – | – | 2 | – | – | 3 |
| Mandible, maxilla, orbit | 3 | – | 1 | 1 | 1 | – | 1 | 2 |
| R Lower lid, L upper lid | 1 | – | 1 | – | – | – | 1 | – |
| Maxilla and scapula | 1 | – | 1 | – | – | – | – | 1 |
| Mandible and testes | 1 | – | 1 | – | – | – | – | 1 |
| Total | 10 | 1 | 5 | 1 | 3 | 0 | 2 | 8 |
| *Stage III patients* | | | | | | | | |
| Abdomen only | 6 | 1 | – | 1 | – | 4 | 3 | 3 |
| Intra-thoracic | 1 | 1 | – | – | – | – | – | 1 |
| Abdomen + other site | 12 | 2 | 3 | 2 | 3 | 2 | 3 | 9 |
| Total | 19 | 4 | 3 | 3 | 3 | 6 | 6 | 13 |
| *Stage IV patients* | | | | | | | | |
| CNS involvement | 3 | – | 3 | – | – | – | 1 | 2 |
| *All stages* | | | | | | | | |
| All sites | 50 | 6 | 12 | 12 | 11 | 9 | 21 | 29 |

All our Stage I patients who died had large tumors with the orbit involved in three, cervical lymph nodes in one and mandible extensively in another. The relationship between gross orbital involvement and poor prognosis in the three cases is not immediately obvious. However, such a relationship is suggested from the three Stage I patients who had gross orbital involvement.

In the present series, Stage I and II patients (localized disease) made up 56% of the total group. In other comparable series, Stage I and II patients comprised 32% (Morrow *et al.*, 1967) and 21% (Ziegler *et al.*, 1969) of their total groups. The higher percentage of patients with localized disease in our series may well be due to understaging in patients who were evaluated prior to the adoption of current staging procedures.

The calculated survival rate in our Stage III patients was better than that reported by Morrow *et al.* (1967) from Uganda. This difference may be due to the smaller number of our Stage III patients (22) as compared to 38 in their series.

Whether surgical reduction of abdominal tumor masses influences survival among Stage III patients, remains in our opinion unresolved. Ngu (1964) and Morrow *et al.* (1967) reported that surgical reduction of abdominal tumor masses appeared to improve the prognosis in Stage III patients. Two of the three Stage III long term survivors in this series had ovarian masses excised. However, five other patients who underwent unilateral or bilateral oophorectomies did not do so well.

Age, in our series, did not seem to influence prognosis. This is at variance with findings by Morrow *et al.* (1967) who noted that in their series younger patients did better than older patients.

The duration of symptoms from reported date of onset to date first seen at any medical facility, within a staging division, bore no relationship to prognosis. This conforms with the findings of Morrow *et al.* (1967). However, the time period given by parents was difficult to verify, since there seemed to be a tendency by parents to under estimate the duration of symptoms.

We are grateful to Dr. Morrow and Dr. Ziegler for useful suggestions and advice, and to Mr. Greenberger and Mr. S. Briandt and his staff for the illustrations. All chemotherapeutic agents used in this study were supplied by the Cancer Therapy Evaluation Branch, Chemotherapy, National Cancer Institute, Bethesda, Maryland.

### REFERENCES

BURKITT, D. AND O'CONOR, G. T.—(1961) *Cancer, N.Y.*, **14**, 258.
CLIFFORD, P.—(1966) *E. Afr. med. J.*, **43**, 179.
HADDOW, A. J.—(1964) *E. Afr. med. J.*, **41**, 1.
MORROW, R. H., PIKE, M. C. AND KISULE, A.—(1967) *Br. med. J.*, iv, 323.
MORROW, R. H., ZIEGLER, J. L.—(1967) Personal communication.
NGU, V. A.—(1964) *Br. J. Cancer*, **19**, 101.—(1968) *W. Afr. med. J.*, **17**, 273.
PIKE, M. C.—(1966) *Lancet*, ii, 856.
ZIELGER, J. L., MORROW, R. H., FASS, L., KYALWAZI, S. K. AND CARBONE, P. P.—(1969) *Cancer, N.Y.*, **26**, 94.